# TLR9 Activation Is Triggered by the Excess of Stimulatory versus Inhibitory Motifs Present in *Trypanosomatidae* DNA

**DOI:** 10.1371/journal.pntd.0003308

**Published:** 2014-11-13

**Authors:** Mélissa Erin Khan, Chloé Borde, Eduardo P.C. Rocha, Véronique Mériaux, Vincent Maréchal, Pedro Escoll, Sophie Goyard, Jean-Marc Cavaillon, Bénédicte Manoury, Noëlle Doyen

**Affiliations:** 1 Institut Pasteur, Département Infection et Epidémiologie, Unité Cytokines & Inflammation, Paris, France; 2 Institut Pasteur, Département Génomes et Génétique, Unité de Génomique Evolutive des Microbes, Paris, France; 3 CNRS UMR3525, Paris, France; 4 Sorbonne Universités, UPMC Université Paris 6, INSERM U1135, Centre d'Immunologie et des Maladies Infectieuses (CIMI), Persistent Viral Infections (PVI) Team, Paris, France; 5 Institut Pasteur, Département Génomes et Génétique, Unité de Biologie des Bactéries intracellulaires, Paris, France; 6 Institut Pasteur, Département Infection et Epidémiologie, Laboratoire des Processus Infectieux à Trypanosomatidés, Paris, France; 7 INSERM U1151, Hôpital Necker-Enfants Malades, Paris, France; New York University, United States of America

## Abstract

DNA sequences purified from distinct organisms, e.g. non vertebrate versus vertebrate ones, were shown to differ in their TLR9 signalling properties especially when either mouse bone marrow-derived- or human dendritic cells (DCs) are probed as target cells. Here we found that the DC-targeting immunostimulatory property of *Leishmania major* DNA is shared by other *Trypanosomatidae* DNA, suggesting that this is a general trait of these eukaryotic single-celled parasites. We first documented, *in vitro*, that the low level of immunostimulatory activity by vertebrate DNA is not due to its limited access to DCs' TLR9. In addition, vertebrate DNA inhibits the activation induced by the parasite DNA. This inhibition could result from the presence of competing elements for TLR9 activation and suggests that DNA from different species can be discriminated by mouse and human DCs. Second, using computational analysis of genomic DNA sequences, it was possible to detect the presence of over-represented inhibitory and under-represented stimulatory sequences in the vertebrate genomes, whereas *L. major* genome displays the opposite trend. Interestingly, this contrasting features between *L. major* and vertebrate genomes in the frequency of these motifs are shared by other *Trypanosomatidae* genomes (*Trypanosoma cruzi, brucei* and *vivax*). We also addressed the possibility that proteins expressed in DCs could interact with DNA and promote TLR9 activation. We found that TLR9 is specifically activated with *L. major* HMGB1-bound DNA and that HMGB1 preferentially binds to *L. major* compared to mouse DNA. Our results highlight that both DNA sequence and vertebrate DNA-binding proteins, such as the mouse HMGB1, allow the TLR9-signaling to be initiated and achieved by *Trypanosomatidae* DNA.

## Author Summary

Distinct laboratory mouse based models have allowed elucidating some of the processes that account for so called resistance or vulnerability to the *Leishmania major* parasite cutaneous inoculation. The outcome ranges from rapid healing – C57BL/6 mice- to progressive nonhealing ones – BALB/c mice. Distinct cell lineages contribute to sense and process molecules derived from the *L. major* parasite. Previous studies revealed a role for intracellular Toll-like receptor 9 (TLR9) in host resistance to *Leishmania major*. *L. major* DNA is involved in innate immune response, since it induces TLR9 signaling and activation of dendritic cells. We were interested to further explore *L. major* DNA sequences focusing on their features as (a) either direct TLR9 agonists or antagonists (b) as well as once partnering with endogenous DNA binding proteins. We more specifically used mouse dendritic cells as sensing cells of *L. major* DNA as well as DNA from other *Trypanosomatidae* in comparison with vertebrate DNA. Overall, the data underscore a counter-selection of TLR9 agonist motifs in vertebrate DNA which is not found in *Trypanosomatidae* DNAs and suggest how TLR9 could discriminate between pathogen and self DNAs, to maintain the cellular integrity.

## Introduction

Toll-like receptors (TLRs) play a crucial role in the recognition of invading pathogens and the subsequent activation of innate immune responses. Several studies revealed a role for intracellular TLR9 in host resistance to protozoan parasites infection including *Trypanosoma brucei*, *Trypanosoma cruzi* and *Leishmania* [1]–[6]. It was also reported that CpG oligonucleotides conveyed protective immunity in lethal murine Leishmaniasis [7]. Previously, it was shown that DNA from *L. major* stimulates TLR9 signaling in dendritic cells (DCs). Importantly, DCs activation did not occur with DNA isolated from vertebrates, suggesting that this activation is specific for *L. major* DNA [8].

Previously the lack of immunostimulatory activity by naked vertebrate DNA has been explained by a combination of several factors such as CpG suppression, CpG methylation, presence of inhibitory motifs and saturable amount of DNA uptake [9], [10]. However, the DNA sequences required for TLR9 activation are controversial, as studies have shown conflicting results regarding the nature of the DNA backbone, the route of DNA uptake and the cell type [11], [12]. Until recently the prevailing paradigm was that TLR9 recognizes unmethylated CpG motifs, which are abundant in bacterial DNA but relatively scarce in mammalian DNA [13]. The idea that stimulatory properties of DNA correlate solely with the lack of CpG methylation may be an over simplification, as hypomethylated mouse DNA fails to activate B cells [14]. Several independent studies have demonstrated that the dependence on CpG motifs for TLR9 activation is restricted to synthetic phosphorothioate (PS) oligonucleotides and that natural phosphodiester (PO) oligonucleotides bind and activate TLR9 via the 2′deoxyribose backbone in a sequence-independent manner [11], [15]–[17]. This result was consistent with the concept that the phosphodiester backbone acts *per se* as a TLR9 agonist. Despite the agonist role of PO backbone, some oligonucleotides sequences were described as stimulatory and others as inhibitory for the TLR9 receptor [10], [13]. Optimal oligonucleotide sequences for TLR9 inhibitory activity were investigated either with PO or PS backbone, revealing a large range of activity in biological assays [17].

It has been proposed that discrimination between microbial and self DNA could be primarily dependent on the colocalization of DNA and TLR9 in endolysosomes [16],[18]. In addition, upon cationic lipids–mediated enhanced endosomal translocation, non-canonical CpG motifs or vertebrate DNA can also trigger cell activation [16], [19] and display TLR9-dependent and -independent activation.

Pathogen-derived DNA may specifically access the TLR9-expressing endosomal compartment in the course of infection whereas host derived DNA may not. Indeed it has been shown that plasmacytoid dendritic cells (pDCs) can respond to self DNA via TLR9 signaling, when self DNA is targeted to the endocytic compartment due to its interaction with circulating auto-antibodies in systemic lupus erythematosus [20] or with the antimicrobial peptide LL37 (cathelicidin antimicrobial peptide from hCAP18) in psoriasis [21]. Other proteins that directly bind to DNA are also involved in endosomal TLR9 activation such as High Mobility Group Box1 (HMGB1), SLPI (Secretory Leucocyte Protease Inhibitor), granulin and CD14 which promote the selective uptake of nucleic acids [22]–[26].

In this work, we enquired on the molecular basis of the differences between *Trypanosomatidae* and vertebrate DNA, both being eukaryotic DNA, regarding TLR9 stimulation. We investigated the uptake of *L. major* and vertebrate DNA in DCs to assess whether the absence of stimulation by vertebrate DNA was due to its limited access to endosomal compartments. Since on one hand similar uptake was observed with the different DNAs and, on the other hand, vertebrate DNA could prevent TLR9 activation by parasitic DNA, we investigated whether their genomic sequence may be involved in their different stimulatory capacity. Therefore we analysed the presence of stimulatory or inhibitory motifs for TLR9, described in previous studies, in both types of DNA.

Furthermore, we investigated whether cofactors in DCs could be involved in the specific activation of TLR9 by parasite DNA. We focused on HMGB1, a mammalian nuclear protein that exhibits a low affinity for linear double-stranded DNA, but which forms stable complexes with unusual or distorted DNA structure [27]. Indeed, HMGB proteins have been demonstrated to act as universal sentinels for nucleic acids [28] and HMGB1 is able to activate TLR9-dependent pathways, when complexed to CpG oligonucleotides [22], [29]. This led us to speculate that HMGB1 might discriminate between vertebrate and parasitic DNA and therefore specifically enhance the activation of DCs by *L. major* DNA.

Our results illustrate that *Trypanosomatidae* and vertebrate DNA differ in the frequency of stimulatory and inhibitory motifs and in their ability to associate with auxiliary factors, such as HMGB1, that may promote the specific activation of TLR9 by parasitic but not vertebrate DNA in DCs.

## Materials and Methods

### Ethics statement

Animals were housed in the Institut Pasteur animal facilities accredited by the French Ministry of Agriculture to perform experiments on mice in appliance of the French and European regulations on care and protection of the Laboratory Animals (EC Directive 86/609, French Law 2001–486 issued on June 6, 2001). The CETEA (Comité d'Ethique pour l'Expérimentation Animale - Ethics Committee for Animal Experimentation) "Paris Centre et Sud" reviewed and approved the animal care and use protocol under the approval number 2012–0059.

### Mice, parasites and reagents

Six to 8 weeks old female C57BL/6 mice were purchased from Charles River Laboratories. TLR9^-/-^ backcrossed to the C57BL/6 background for10 generations were provided by S. Akira (Osaka University, Osaka, Japan). All mice were bred in our facilities and housed under specific pathogen-free conditions.

Promastigotes of *L. major* LV39, *T. vivax, T. cruzi, T. brucei* were propagated *in vitro* in adapted medium supplemented with 10% of Foetal Calf Serum. Genomic DNA from vertebrate kidney and lymphnodes purified cells and from *L. major, T. vivax, T. cruzi, T. brucei* parasites were prepared by proteinase K and RNase digestion followed by phenol/chloroform extraction and ethanol precipitation. Vertebrate and parasite DNA mean purified and naked DNA whereas all complexed DNA are purified DNA in which we added different factors. We used DOTAP (cationic lipid N-(2,3-Dioleoyloxy-1-propyl) trimethylammonium methyl sulfate) (Sigma-Aldrich), CpG ODN type B 1826 (TCCATGACGTTCCTGACGTT), CpG ODN type A 2216 (5′-GGGACGATCGTG-3′) (Sigma-Proligo), ODN 2088 (5′-TCCTGGCGGGGAAGT-3′) (Eurogentec), imidazoquinoline Cl-097 (InvivoGen), DNase I (2 U/ml) (BioLabs), DNAse II (1100 U/ml) and glycyrrhizin (Sigma-Aldrich). HMGB1 was kindly provided by Dr. V. Maréchal (Centre de recherche des Cordeliers, France) [30]. Residual lipopolysaccharide, quantified using the E-toxate assay (Sigma) was less than 100 fg per microgram of HMGB1.

### Cell culture

Bone marrow (BM) cells were isolated by flushing mice femurs and tibias with PBS. After treatment with Red Blood Cells lysis buffer (Sigma-Aldrich), BM cells were cultured in complete RPMI 1640 supplemented with GM-CSF from J558L cell line supernatant [31]. At day 8, 75–80% of cells are BM derived dendritic cells (BMDCs) CD11c^+^CD11b^+^.

Gen2.2 is a plasmacytoid cell line pDC provided by a leukaemia patient [32]. Briefly, they grow on a murine fibroblast feeder cell line MS5 in RPMI, supplemented with 10% FCS, 1% L-glutamine, non-essential amino acids, gentamicin and 0.2% sodium pyruvate.

### Stimulation of BMDCs and Gen2.2

BMDCs or Gen2.2 were cultured in 6-well plates (3×10^6^) using complete RPMI 1640. Cells were activated for 6 h with *L. major, T. vivax, T. cruzi, T. brucei* or vertebrate (mouse, pig, sheep or human) genomic DNA (40 to 2.5 µg), 0.25 µg CpG 1826, 5 µg CpG 2216 or 0.25 µg Cl-097. When indicated, stimulation was done with DNA complexed with DOTAP (10 µg for 2.5–5 µg of DNA) or with HMGB1 (1 µg), SLPI (20 µg) or LL37 (2 µg). Before being added to the cells, DNA was incubated with DOTAP or the various peptide/protein 20 min at RT. Each factor was tested alone to induce no stimulation by itself. In some experiments, cells were treated with chloroquine at 20 µM for 1 h before activation. For DNA competition experiments, cells were incubated with both parasite and vertebrate DNAs. Supernatants and cells were harvested for ELISA or RNA extraction.

### ELISA

IL-6 and TNFα were quantified in cell culture supernatants using the BD OptEIA TNFα and IL-6 ELISA set (BD Biosciences). All ELISA procedures were performed according to the manufacturer's protocol.

### RNA extraction and quantitative RT-PCR

RNA was extracted from BMDCs or Gen2.2 using a microRNeasy extraction kit (Qiagen). A trace of genomic DNA was removed by RNAse free-DNAse set. RNA (2 µg) was reverse transcribed using (200 U) Moloney murine leukemia virus reverse transcriptase (SuperScript II, Invitrogen). Subsequent real time PCR was performed on Step One Plus (Applied Biosystems) using Taq polymerase or SYBER green (Taq-Man Universal or SYBER Green PCR master mix, Applied biosystems).

### Flow cytometry

Flow cytometric data were acquired on a four-color FACS Calibur cytometer (BD Biosciences) and analysis was done with Cell Quest Pro software. For surface phenotyping, the following antibodies were used: anti-human CD45RA-FITC (MEM-56), CD11c-APC (BU15), HLA-DR-FITC (LT-DR), CD123-PE (9F5); anti-mouse CD11b-PE (M1/70), CD11c-APC (HL3) (BD Bioscience). Uptake of propidium iodide-stained DNA in CD11c-APC cells was performed on FL3 channel.

### Immunofluorescence

For DNA uptake experiments, BMDCs were plated on a µ-slide 8 well ibiTreat (ibidi) then stimulated for 1 h with *L. major* or mouse DNA. DNA was stained overnight with propidium iodide (PI) and precipitated in ethanol. Cells were fixed with 2% paraformaldehyde for 20 min at RT. Images were taken using a Leica SP5 scanning confocal microscope with an ×63 oil objective. DAPI and propidium iodide signal was acquired following respective excitation at 405 nm and 561 nm and respective emission at 420/465 nm and 577/690 nm, in sequential captures with optical sections of 0.4–0.8 µm. All microscope parameters were kept constant between experiments. Icy software (version 1.3.6.0) was used for merge analysis and image presentation [33]. ImageJ software (version 1.47q) was used to calculate the average DNA uptake into cells.

### Analysis of HMGB1-DNA complexe by western blot

Gel retardation experiments were performed as previously described [34] with the following modifications. Increasing amounts of HMGB1 protein (0.125 µg to 2.5 µg) were incubated for 20 min with 250 ng of sonicated parasite or vertebrate DNA in a buffer containing 0.15 M NaCl, 10 mM Tris-HCl pH 7.6. The complexes were loaded on a 1% agarose-TBE gel. Following electrophoretic migration the gels were either immediately transferred onto PVDF (polyvinyl difluoride) membranes (Amersham Biosciences) or stained in a solution of 0.5 µg/ml ethidium bromide (EtBr) for 45 min before transfer. EtBr did not stain the gel before transfer to avoid diffusion of free HMGB1.

Then membranes were probed with antibodies against HMGB1 (Abcam, 1∶1000).

### Subcellular distribution of HMGB1

BMDCs (3×10^6^) were stimulated with CpG 1826 (1 µg), *L. major* or vertebrate DNA (20 µg) for 30 min and 1 h. The cells were lysed in buffer (20 mM TrisHCl (pH 7.4), 10 mM NaCl, 3 mM MgCl_2_) with anti-proteases for 30 min at 4°C. After centrifugation at 3000 rpm, the cytoplasmic fraction was harvested and the nuclear fraction extracted with the buffer from Subcellular Proteome Extraction kit (Calbiochem). The fractions were analysed by Western Blotting, run in a 10% SDS-PAGE, probed with antibodies against HMGB1 (1∶1000, Abcam), actine (1∶10000, Sigma), histone H3 (1∶5000, Abcam) and revealed with an anti-rabbit or anti-mouse immunoglobulin-horseradish peroxidase conjugates (1∶10000, Serotec). Quantification was performed with ImageJ software.

### DNAse activity assay

*L. major* or vertebrate DNA (1 µg) was incubated with DNAse I or II at different concentrations for 30 min at 37°C, and 10 min at 75°C in buffer (10 mM Tris-HCl, 2.5 mM MgCl_2_, 0.5 mM CaCl_2_, pH 7.6 or 150 mM Na acetate-HCl, 5 mM EDTA pH 4.5 respectively). With cytoplasmic extract (up to 5 µg), DNA was incubated for 2 h at 37° and 10 min at 75°. Cleavage products were analysed by 0.7% agarose gel electrophoresis, stained with EtBr.

### Genome analysis

The genomes analysed are referenced in GenBank Assembly database as: GCA_000002725.2 for *Leishmania major* genome strain Friedlin by the Friedlin Consortium, GCA_000001635.4 for mouse (Mus musculus) genome by the Genome Reference Consortium Mouse and GCA_000001405.13 for human (Homo sapiens) genome by the Genome Reference Consortium Human. The *Trypanosomatidae* genomes referenced *T. cruzi* strain CL Brener Esmeraldo-like, *T. brucei* strain TREU 927 and *T. vivax* strain Y486 (2013-01-16 versions for all) were taken from *Trypanosomatidae* database TritrypDB (http://tritrypdb.org/tritrypdb/) [35]. All bioinformatic analyses, including motif counts and determination of genome size, were made from this same dataset. The different motifs were searched in each chromosome or in the whole genome with in-house software (wcount). The motif is represented using the IUPAC (International Union of Pure and Applied Chemistry) nucleotide ambiguity code.

### Statistical analysis

Statistical significance was tested using Prism 5.0 (GraphPad Software) by Mann-Whitney test (for cells activation and chromosomes analysis) and Wilcoxon signed-rank test (for genomes analysis, to test whether rO/E is different from 1). Error bars in all figures represent SEM, with the midline representing the mean value.

### Genomic or proteins sequences accession numbers or ID

Here is the list of accession numbers/ID numbers for genomes mentioned in the text:

- GCA_000002725.2 for Leishmania major genome strain Friedlin by the Friedlin Consortium
- GCA_000001635.4 for mouse (Mus musculus) genome by the Genome Reference Consortium Mouse
- GCA_000001405.13 for human (Homo sapiens) genome by the Genome Reference Consortium Human.

Genome sequences from the primary assemblies for the other *Trypanosomatidae* organisms were obtained in TriTrypDB (http://tritrypdb.org/tritrypdb/) and are publicly accessible in the cited link in Data Summary/Genomes and Data Types. The genomic sequences are available under:

- the organism ID tcruCLBrenerEsmeraldo-like for the *Trypanosoma cruzi* strain CL Brener Esmeraldo-like
- the organism ID tbruTREU927 for *Trypanosoma brucei* strain TREU 927
- the organism ID tvivY486 for *Trypanosoma vivax* strain Y486

The proteins studied in the text are listed below, publicly accessible in Uniprot database:

- murine TLR9: Q9EQU3
- murine SLPI: P97430
- murine HMGB1: P63158
- human LL37, part of hCAP-18: P49913

## Results

### TLR9-dependent specific activation of dendritic cells by *Trypanosomatidae* DNA

**Figure 1:**
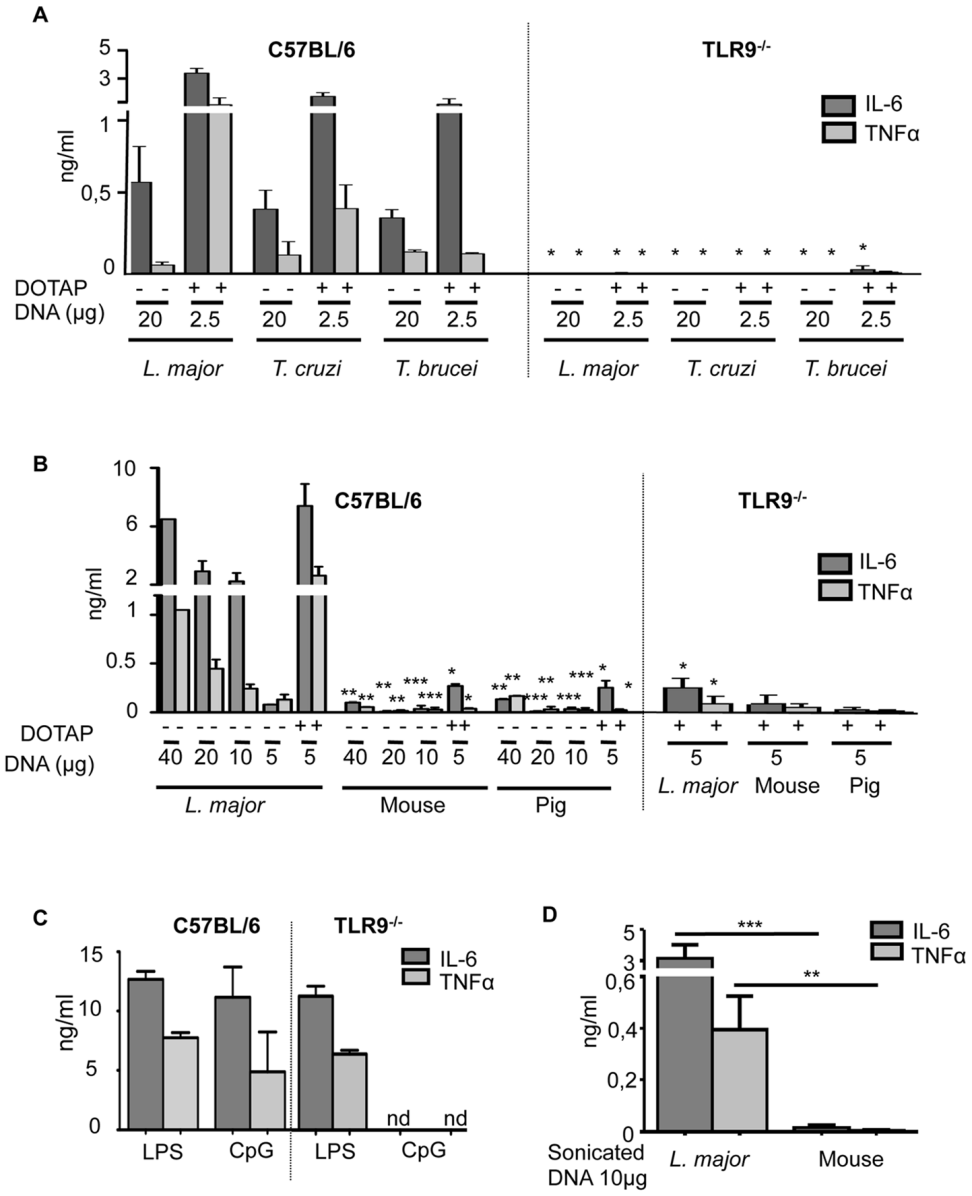
TLR9-dependent activation of BMDCs specific to *Trypanosomatidae* DNA. C57BL/6 or TLR9^-/-^ BMDCs were stimulated *in vitro* either with *Trypanosomatidae* or vertebrate DNA for 6 hours (**A–C**) IL-6 and TNFα production was measured by ELISA in the supernatants of stimulated BMDCs for 6 h (**A**) with *Trypanosomatidae* DNA alone or complexed with DOTAP, (**B**) with different concentrations of *L. major* or vertebrate (mouse or pig) DNA alone or complexed with DOTAP, (**C**) with CpG 1826 (0,25 µg/ml) and LPS (100 ng/ml) as controls, (**D**) with *L. major* or mouse DNA sonicated in 200 base pair (bp) fragments. The data represent the mean and SEM of three independent experiments. Significant differences were found between C57BL/6 and TLR9^-/-^ in A, B, C (*, p<0.05) and between *L. major* and vertebrate DNA (mouse or pig) in C and D (*, p<0.05; **, p<0.01; ***, p<0.001).

It was previously shown that *L. major* DNA could activate cytokine expression in BMDCs (bone-marrow derived DCs) from C57BL/6 mice but had no effect on BMDCs from TLR9-deficient mice. We show here that this property is shared by other *Trypanosomatidae* DNA (*T*) including *T. cruzi, T. brucei* and *T. vivax* (Figure 1A and Figure S1).

When *Trypanosomatidae* DNAs are complexed with DOTAP to enhance the endosomal translocation of DNA, a 8-fold lower amount of DNA induces higher cytokine production by BMDCs in comparison to naked DNA (Figure 1A). While we observe an increase in the expression of cytokines (IL-6 and TNFα) proportional to the amount of *L. major* DNA added, we do not detect either TLR9 -dependent or -independent activation with different vertebrate DNAs until 40 µg/ml and at that point we detect minimal activation. In addition the activation of DCs by vertebrate DNA complexed with DOTAP is 10 times lower than that obtained with 4 times less DNA from *L. major* (Figure 1B and Figure S2A). We used CpG (0.25 µg/ml) and LPS (100 ng/ml) as controls to test the capacity of the cells to be activated by stimuli requiring TLR9 or not (Figure 1C and Figure S1).

Each mouse chromosome is at least 20-fold longer than any *L. major* chromosome. Therefore, we investigated whether the low level of activation by vertebrate DNA could be due to its size, which may prevent its uptake by the cell. *L. major* DNA sonicated into 200 to 500 base pair fragments (Figure S2B) induced the production of proinflammatory cytokines, while sonicated vertebrate DNA did not (Figure 1D), demonstrating that chromosomal size discrepancy did not account for the differences between *L. major* and vertebrate DNA in TLR9 activation.

**Figure 2:**
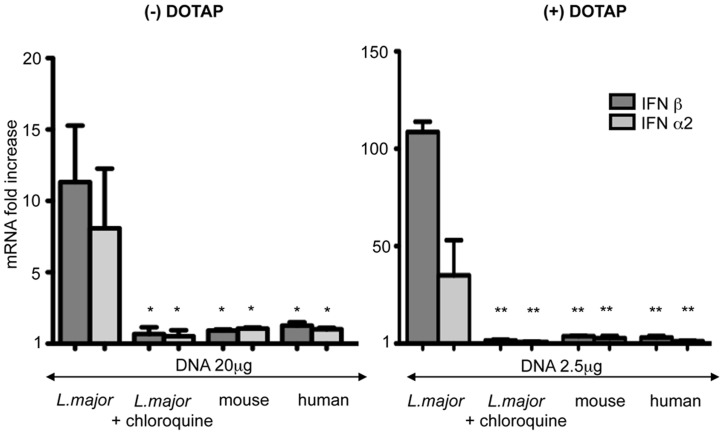
Stimulation of human plasmacytoid dendritic cells by *L. major* and vertebrate DNA. Human plasmacytoid cell line (Gen2.2) was stimulated by *L. major*, mouse and human DNA alone ***(left)*** or complexed with DOTAP ***(right)***. In the indicated lanes, cells were treated with chloroquine (20 µM) before *L. major* stimulation. Expression of the indicated cytokines was determined by real time RT-PCR. The mRNA expression levels were normalized to the expression of the HPRT gene and calculated as the n-fold difference with the expression in unstimulated cells. The results represent the mean and SEM of three independent experiments (*p<0,05, **p<0,01).

To address whether human TLR9 could also discriminate between *L. major* and vertebrate DNA, we investigated TLR9 signaling in human plasmacytoid DCs (pDCs). Because of their low frequency in human blood, we used a human pDCs cell line GEN2.2 CD123^+^ HLA-DR^+^, derived from leukemic pDCs [32], which were activated by TLR9 and TLR7 agonists (CpG and Cl-097) (Figure S3). Only *L. major* DNA induced the increase of IFNα2 and IFNβ in pDCs (Figure 2). No comparable activation was observed with the same quantity of vertebrate DNA, even in the presence of DOTAP. The activation was impaired by chloroquine treatment, which inhibits the endosomal acidification necessary for TLR9 activation. Thus, in bone marrow derived DCs from mouse and in a human plasmacytoid cell line that have been only investigated, we showed that *L. major* DNA induced TLR9 signaling at least 10 times more efficiently than vertebrate DNA.

### Similar cellular uptake of *Trypanosomatidae* and vertebrate DNA by BMDCs

**Figure 3:**
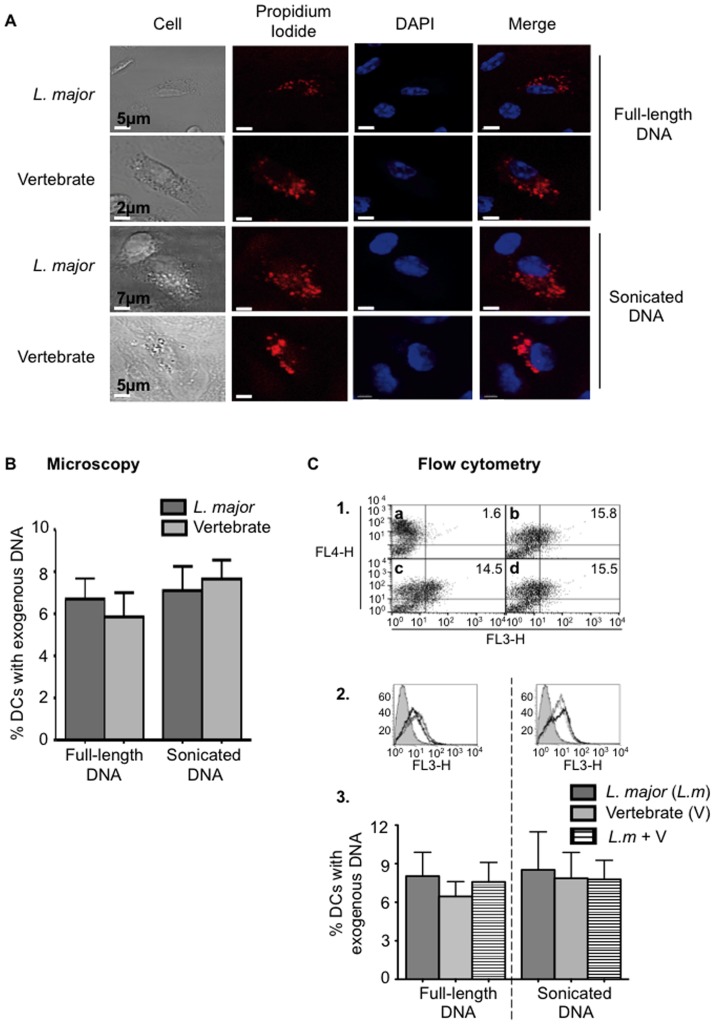
Comparative analysis of mouse and *L. major* DNA cell uptake. Analysis by confocal microscopy (**A, B**) and flow cytometry (**C**) of BMDCs stimulated 1 h by 10 µg of full-length DNA or sonicated DNA (200 bp) from *L. major* or mouse labelled with propidium iodide (PI) (red). (**A, B**) Nuclei are stained with DAPI (blue). Quantification was performed by counting 250–300 BMDCs per condition per experiment. (**C**) Internalized DNA was detected in FL3 channel in BMDCs labelled with an anti-CD11c APC (FL4 channel). **1**) Dot plots represent BMDCs alone (**a**) or incubated with *L. major* DNA (**b**), vertebrate DNA (**c**) or both DNAs (**d**). The percentage of BMDCs PI^+^CD11c^+^ (respectively in FL3 and FL4 channels) is indicated for each dot plot. **2**) Filled and open histograms represent unstimulated or stimulated BMDCs with PI stained DNA. *L. major* DNA (*L.m*), vertebrate DNA (V) and *L. major* (10 µg) with vertebrate DNA (10 µg) (*L.m*+V) are represented respectively with a thin, solid and a dashed line. **3**) Percentage of BMDCs with exogenous DNA. In the experience of uptake competition, only *L. major* DNA was stained with PI. The data represent three independent experiments: one representative for A C1 and C2 and the mean and SEM in B and C3.

Differential DNA uptake could account for the difference for TLR9 signaling between *L. major* and vertebrate DNAs. To compare their uptake in BMDCs, both purified DNAs were labeled with propidium iodide (PI) and added to the cells. To avoid PI diffusion, the process of DNA uptake was analysed after one-hour incubation with the cells by confocal microscopy (Figure 3A and 3B) or flow cytometry (Figure 3C). We detected 6–10% of BMDCs containing exogenous full-length DNA with both techniques. Surprisingly we found the same proportion of DNA-containing BMDCs when exogenous sonicated DNA was used (Figure 3A, 3B and 3C). Thus, the uptake of *L. major* and vertebrate DNA is not significantly different. We also investigated whether *L. major* and vertebrate DNAs could compete for cellular uptake and tracked their internalization in BMDCs by flow cytometry. No difference in L. major DNA uptake was observed in presence of vertebrate DNA (Figure 3).

### Vertebrate DNA is more sensitive to DNase I and II than *L. major* DNA

**Figure 4:**
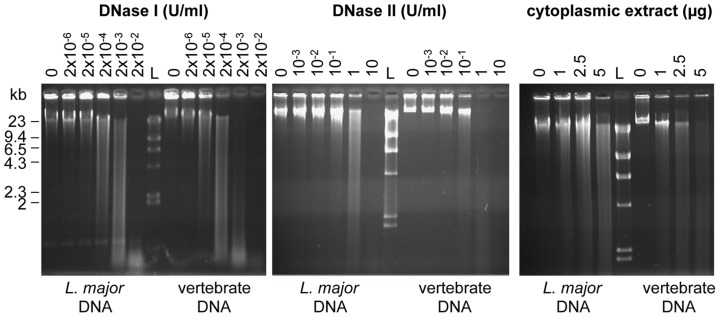
*L. major* and vertebrate DNA differ in their DNAse sensitivity. DNA degradation was analysed by electrophoresis on a 0.7% agarose gel, stained by EtBr. Increasing amounts of DNAse I ***(left)***, DNase II ***(middle)*** or cytoplasmic extract from C57BL/6 BMDCs ***(right)*** was added to a same amount of full-length genomic *L. major* or vertebrate DNA (1 µg). L stands for the Kb ladder (λDNA-Hind III). The data represent one representative of three experiments.

Degradation of exogenous DNA by DNAses may be a limit to TLR9 activation. Since the DNase content is much higher in phagocytic cells, such as DCs, than in other cells, we compared the relative sensitivity of *L. major* and mouse genomic DNA to increasing concentrations of both DNase I or II (Figure 4). DNase I and II nucleases are usually involved in the digestion of DNA that originated outside the nucleus [36]. We observed that the complete degradation of *L. major* DNA requires ten times more purified DNase I or II or 2 times more cytoplasmic extract than for vertebrate DNA (Figure 4). Thus, *L. major* DNA is intrinsically more resistant to DNase than vertebrate DNA suggesting that the parasitic DNA could persist longer in the cells.

### Vertebrate DNA prevents the immunostimulatory activity of *L. major* DNA

**Figure 5:**
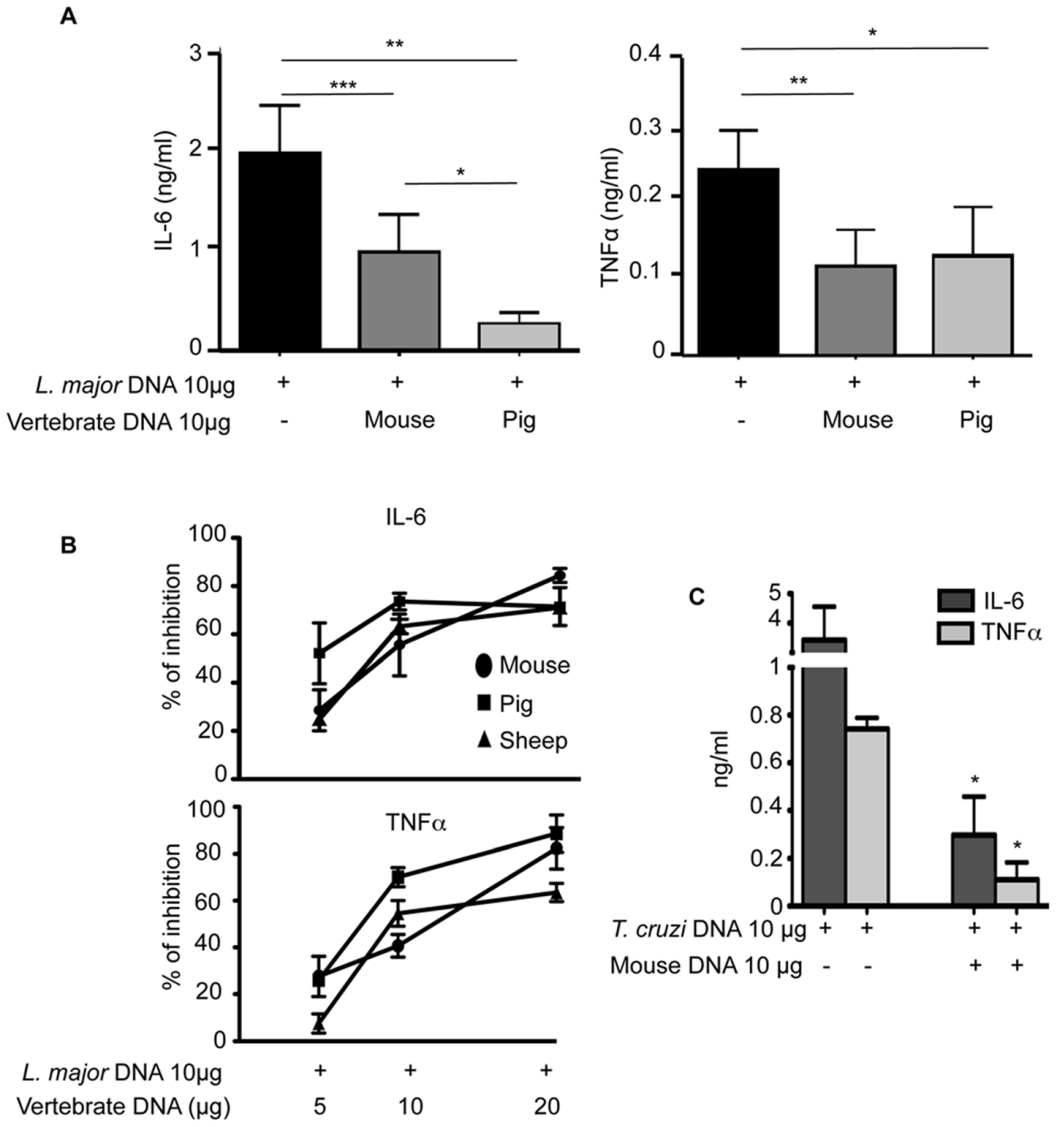
Competition with vertebrate DNA prevents the immunostimulatory activity of *L. major* DNA. C57BL/6 BMDCs were stimulated *in vitro* for 6 h with 10 µg of *L. major* DNA (**A–B**) or *T. cruzi* DNA (**C**), alone or with 10 µg of vertebrate DNA. Cytokines production was measured by ELISA in the supernatants of cultures. (**A and C**) The data represent the mean and SEM of three independent experiments. Significant differences are indicated (*, p<0.05; **, p<0.01; ***, p<0.001). (**B**) The percentage of inhibition of BMDCs activation obtained by adding increasing amount of vertebrate DNA (mouse, pig or sheep) to *L. major* DNA. Percentage (%) of inhibition  =  [100-{cytokines production by *L. major* with vertebrate DNA/cytokines production by *L. major* DNA alone}]×100. The results represent the mean and SEM of three independent experiments.

Given the different properties of *L. major* and vertebrate DNA regarding TLR9 activation and DNase sensitivity, we wondered whether these DNA might be in competition for TLR9 activation. The addition of different vertebrate DNA (mouse or pig) to *L. major* DNA inhibited the activation of BMDCs induced by *L. major* DNA alone. Indeed, the production of IL-6 and TNFα by BMDCs is significantly reduced (Figure 5A). This inhibition increases in relation to the concentration of vertebrate DNA (Figure 5B). The percentage of inhibition reached approximatively 30 to 50% depending on the species with an identical amount of vertebrate and *L. major* DNA and reaches up to 70% to 85% in the presence of a two fold excess vertebrate DNA. It should be noted a slightly higher inhibition with pig versus mouse DNA (10 µg) characterized by a lower production of IL-6 (Figure 5A) but not statistically different with 20 µg of DNA (Figure 5B). Neither toxic effect nor inhibition of LPS activation was observed with high concentration of DNA (20 or 40 µg) (Figure 1B and Figure S4), indicating that this inhibition was specific of the TLR9 activation pathway. We also noticed that sonicated vertebrate DNA and inhibitory oligonucleotide inhibited the activation induced by *L. major* DNA (Figure S4). Since sonicated vertebrate DNA did not cause cellular activation (Figure 1D) while exhibiting an inhibitory capacity (Figure S4) we could conclude that full-length DNA and degraded vertebrate DNA have the same properties. Importantly this would suggest that the higher sensitivity of vertebrate DNA to cellular DNAses should not interfere with its inhibitory capacity.

Additionnal experiments demonstrated that mouse DNA could also inhibit the activation of BMDCs induced by *T. cruzi* DNA alone (Figure 5C). The percentage of inhibition was about 90% when vertebrate DNA was added to *T. cruzi* parasite DNA. Inhibition by vertebrate DNA may therefore be generalized to other *Trypanosomatidae* DNAs.

The inhibition by naked vertebrate DNA could reflect a competition between both DNAs for TLR9 activation and suggest that discrimination between *Trypanosomatidae* and vertebrate DNAs involved their genomic sequences.

### Different distribution of inhibitory and activating sequences between *L. major* and vertebrates

**Figure 6:**
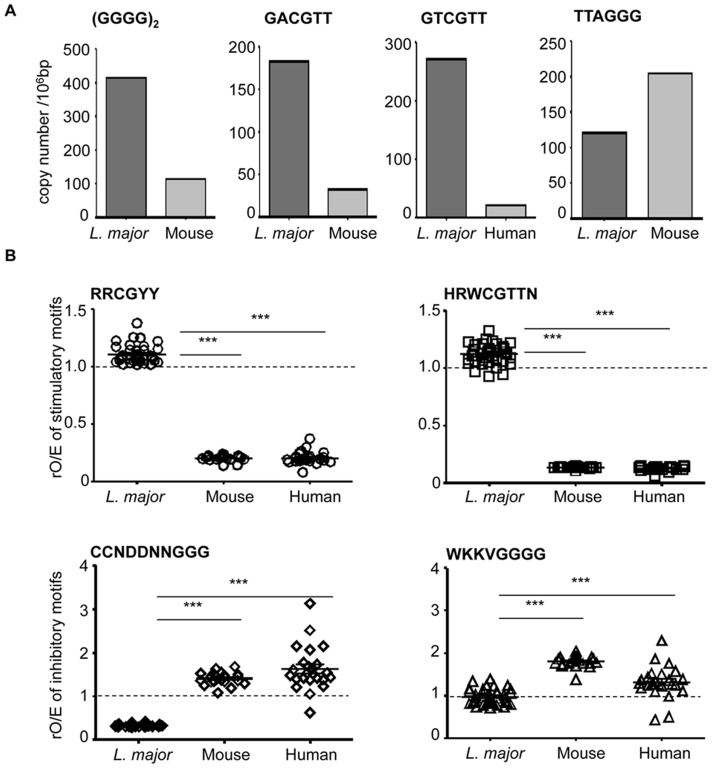
Selection for inhibitory and against stimulatory TLR9 motifs in vertebrate genome. (**A**) Poly G_8_ (GGGG)_2_, stimulatory GACGTT and inhibitory TTAGGG motifs were quantified in *L. major* and mouse genomes. Stimulatory GTCGTT motif was quantified in *L. major* and human genomes. The datas are represented as copy number per 10^6^ bases pair ( = copy number/genome size (bp)*10^6^). (**B**) Stimulatory (RRCGYY and HRWCGTTN) and inhibitory (WKKVGGGG and CCNDDNNGGG) motifs were quantified in each *L. major*, mouse or human chromosome. They are represented as the ratio of observed/expected sequences rO/E, as indicated in Table 1. For each chromosome, the ratio is represented by a single symbol. Significant differences between *L. major* and vertebrate chromosomes are indicated (***, p<0.001). The dotted line represent the ratio of observed/expected rO/E sequences which is 1, when no selection pressure is exerted on the genome in a neutral environment.

We analyzed the genomic frequency of motifs affecting the activation or inhibition of TLR9 in *L. major* and vertebrate (mouse and human) DNA. 3′extension with polyG reduces nuclease sensitivity [37]. In agreement with the greater resistance of *L. major* DNA to DNase, we found that the relative frequency of polyG_8_ motif (represented here as (GGGG)_2_) was 4 times larger in the genome of *L. major* than in the mouse genome (Figure 6A and Table S1).

Additionally, we searched for the CpG motifs GACGTT or GTCGTT, respectively defined as the mouse and human optimal TLR9 activating motifs [13], [38]. These stimulatory motifs are 6 and 13 times more frequent in *L. major* than in mouse and human genomes respectively. In contrast, the inhibitory telomeric motif TTAGGG [39] is 2 times less frequent in the *L. major* genome than in the mouse genome (Figure 6A and Table S1).

To extend these observations, we examined all the combinations around the dinucleotide CpG in the canonical motif (RRCGYY) and other motifs that are analogous to oligonucleotides previously described as activators (HRWCGTTN) [10], [13] that are found in every class of CpG. We also investigated combinations around G-rich sequence described to have inhibitory properties (WKKVGGGG) and the optimal TLR9 inhibitory sequence CCNDDNNGGG [10], [17]. Table 1 shows the total number of each set of motifs in the different genomes. We also computed the number of expected motifs given the frequency of each nucleotide and genome size. From these data, we obtained the ratio of observed over expected motifs (rO/E) for each genome. For stimulatory motifs RRCGYY and HRWCGTTN, the ratio is around 1 in *L. major* genome. This ratio is lower in the mouse and human genomes (respectively 0.20 and 0.13). The TLR9 inhibitory motifs CCNDDNNGGG and WKKVGGGG are rare in the *L. major* genome (rO/E of 0.33 and 0.93 respectively). In human and mouse genomes inhibitory motifs are more frequent than expected (between 1.3 and 1.8).

**Table 1:** Analysis for stimulatory and inhibitory motifs in *Trypanosomatidae* and vertebrate genomes.

	Organism	Frequency	Expected number	Observed number	rO/E
**Stimulatory motif**					
**RRCGYY**	*L. major*	5.63.10^−3^	184500	197597	1.07
	*T. cruzi*	4.23.10^−3^	137439	105362	0.77
	*T. brucei*	3.31.10^−3^	87665	82685	0.94
	*T. vivax*	4.56.10^−3^	103513	70098	0.68
	Mouse	2.76.10^−3^	7607250	1490164	0.20
	Human	2.76.10^−3^	8875125	1665432	0.19
**HRWCGTTN**	*L. major*	5.04.10^−4^	16531	18555	1.12
	*T. cruzi*	6.92.10^−4^	22496	17819	0.79
	*T. brucei*	8.02.10^−4^	21258	18976	0.89
	*T. vivax*	6.47.10^−4^	14710	13428	0.91
	Mouse	8.50.10^−4^	2345140	309431	0.13
	Human	8.50.10^−4^	2735997	347034	0.13
**Inhibitory motif**					
**CCNDDNNGGG**	*L. major*	1.19.10^−3^	39055	12726	0.33
	*T. cruzi*	6.51.10^−4^	21165	8478	0.40
	*T. brucei*	3.82.10^−4^	10118	7873	0.78
	*T. vivax*	7.65.10^−4^	17372	5267	0.30
	Mouse	2.55.10^−4^	703493	936758	1.33
	Human	2.55.10^−4^	820742	1183790	1.44
**WKKVGGGG**	*L. major*	6.48.10^−4^	21254	19451	0.92
	*T. cruzi*	4.17.10^−4^	13557	10625	0.78
	*T. brucei*	2.76.10^−4^	7312	9605	1.31
	*T. vivax*	4.71.10^−4^	10691	5305	0.50
	Mouse	2.00.10^−4^	552602	944031	1.71
	Human	2.00.10^−4^	644703	787326	1.22

Stimulatory motifs (RRCGYY for [purin-purin-C-G-pyrimidin-pyrimidin]; HRWCGTTN for [(notG)-purin-(A or T)-C-G-T-T-any base] and inhibitory motifs (WKKVGGGG for [(A or T)-(G or T)-(G or T)-(A or C or T)-G-G-G-G]; CCNDDNNGGG for [C-C-x-(notC)-(notC)-x-x-G-G-G], where × stands for any base) were searched in whole genomes. For each motif, the counted number was indicated as the observed number. The expected motif number was calculated as (motif frequency × genome size). The motif frequency corresponds to the product of frequency for each nucleotide, which is different from one genome to another (Table S1). The rO/E corresponds to the ratio between the observed number and the expected number of the motifs.

We analyzed the distribution of stimulatory and inhibitory motifs in each of the 36 chromosomes of *L. major* and then compared it statistically with that of the 21 mouse chromosomes and 24 human chromosomes (Figure 6B). The rO/E values for the activating motifs RRCGYY and HRWCGTTN were very similar among chromosomes of the same genome. The rO/E values for the inhibitory motifs CCNDDNNGGG and WKKVGGGG were very similar in the 36 chromosomes of *L. major* (<1), but more diverse among mouse and human chromosomes. Importantly, rO/E values for inhibitory motifs were systematically and very significantly lower in *L. major* than in human and mouse genomes (p<0.0001 for both, Mann-Whitney test). Conversely, the rO/E values of stimulatory motifs were systematically and significantly higher in *L. major* than in human and mouse genomes (p<0.0001 for both, Mann-Whitney test). Thus, in contrast to human and mouse genomes that have counter-selected TLR9-stimulatory motifs and over-represented TLR9-inhibitory motifs, our results show that in *L. major* genome, there is no selection of motifs affecting the activation of TLR9.

### Similar distribution of inhibitory and stimulatory motifs in *L. major* and *Trypanosomatidae* genome

**Figure 7:**
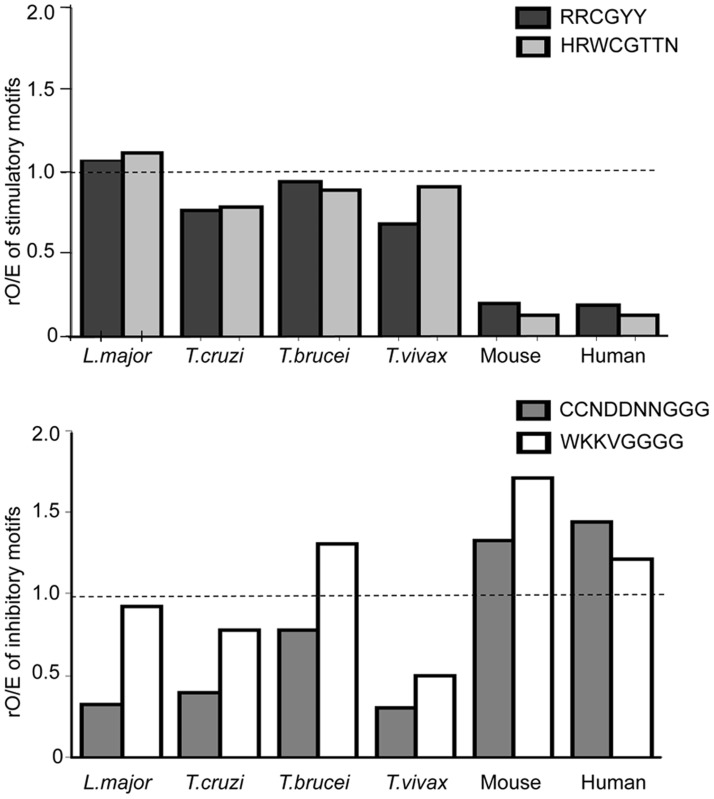
The representation of stimulatory and inhibitory motifs in *L. major* genome is shared by other *Trypanosomatidae* genomes. The data represent the ratio of observed/expected number rO/E for each motif [stimulatory (RRCGYY and HRWCGTTN) or inhibitory (WKKVGGGG and CCNDDNNGGG)] from the analysis of *Trypanosomatidae* complete genomes (Table 1). The dotted line represent the ratio of observed/expected rO/E sequences which is 1, when no selection pressure is exerted on the genome in a neutral environment.

We wondered whether these observations could be extended from *L. major* to other *Trypanosomatidae* DNA. The ratios rO/E for both stimulatory motifs RRCGYY and HRWCGTTN are identical and reach 1.1, 0.8, 0.9 respectively in *L. major, T. cruzi* and *T. brucei*; for *T. vivax* the ratios are 0.68 and 0.91 (Figure 7 and Table 1). However, these ratios are always higher in *Trypanosomatidae* genomes than in vertebrate genomes. In contrast, the ratio of the two inhibitory motifs is more variable between *Trypanosomatidae* genomes. The ratios are between 0.3 and 0.9 for *L. major*, *T. cruzi* and *T. vivax* genome and slightly higher than 1 only for *T. brucei.*

As the genomes of those *Trypanosomatidae* parasites have not all been assembled yet, we were not able to analyze the motifs distribution on their chromosomes. Therefore, we calculated the ratios S/I between each stimulatory and inhibitory sequence (Table 2). The ratio between the canonical stimulatory motif RRCGYY and each inhibitory motif is slightly higher in *L. major* than *T. cruzi* and higher in *T. cruzi* than T. *brucei*. Interestingly, the order of the ratios matches well the order of activation. With the second stimulatory motif HRWVGTTN, the ratio S/I are around 2 but they represent around 20% of the canonical motif RRCGYY, except in *L. major* genome (10%). All the S/I ratios in *Trypanosomatidae* genome are 3 to 10 times higher than those in vertebrate genome (Table 2).

**Table 2:** Ratio between stimulatory and inhibitory motifs in *trypanosomatidae* and vertebrate genomes.

S/I	*L. major*	*T. cruzi*	*T. brucei*	*T. vivax*	Mouse	Human
**A/C**	15.5	12.4	10.5	13.3	1.6	1.4
**A/D**	10.2	9.9	8.6	13.2	1.6	2.1
**B/C**	1.5	2.1	2.4	2.5	0.3	0.3
**B/D**	1.0	1.7	2.0	2.5	0.3	0.4
**A- RRCGYY**	197597	105362	82685	70098	1490164	1665432
**B- HRWCGTTN**	18555	17819	18976	13428	309431	347034
**C- CCNDDNNGGG**	12726	8478	7873	5267	936758	1183790
**D- WKKVGGGG**	19451	10625	9605	5305	944031	787326

The table shows the S/I ratio that corresponds to (number of stimulatory motif/number of inhibitory motif) calculated for each motif and genome. The number of motifs observed in each genome is reported as in Table 1, A and B corresponding to the stimulatory motifs and C and D to the inhibitory ones. The S/I ratios are significantly higher in *trypanosomatidae* DNA than in mouse DNA, according to Wilcoxon test (p<0.5).

Overall, this analysis demonstrates that the contrast observed in the genomic frequency of inhibitory and stimulatory motifs between *L. major* DNA and human and mouse genomes is shared with the other *Trypanosomatidae* DNA.

### HMGB1 enhances TLR9-dependent dendritic cell activation by *L. major* DNA

Different factors such as cationic peptides can interact with DNA and facilitate its access to the endosomal TLR9 receptor. We observed an overall increase in the expression of cytokine mRNA in BMDCs when *L. major* DNA was complexed with HMGB1 proteins or with cationic peptides such as LL37 and SLPI (secretory leucocyte protease inhibitor) (Figure S5). We focused on HMGB1 since it could mediate TLR9 activation by DNA at a low concentration.

**Figure 8:**
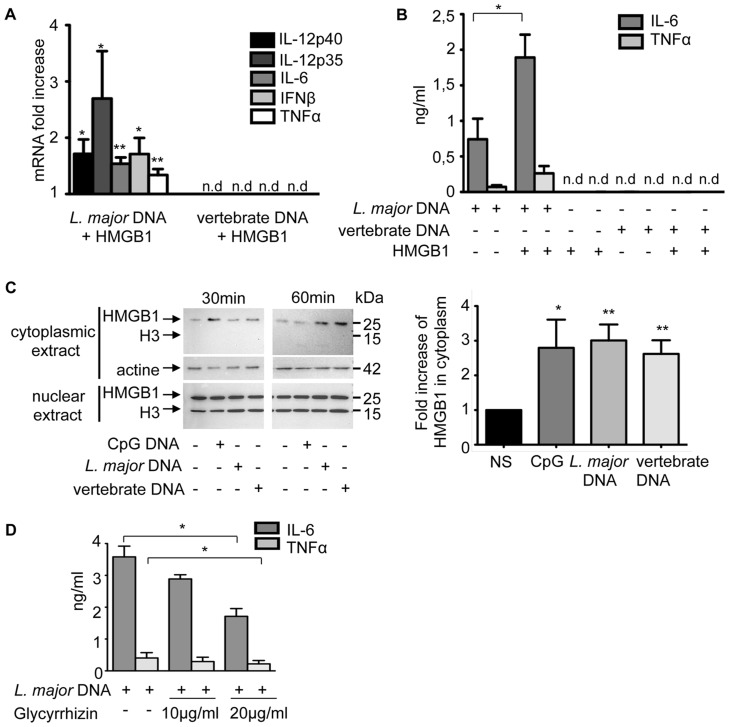
The contribution of HMGB1 to TLR9 activation. C57BL/6 BMDCs were stimulated with *L. major* or vertebrate DNA (20 µg) complexed with rHMGB1 (1 µg) or alone (as control and reference) for 6 h. (**A**) Cytokines mRNA were detected by real-time PCR. The data are expressed as the n-fold increase with the expression in stimulated BMDCs by *L. major* or vertebrate DNA alone. The mRNA expression levels were normalized to the expression of the HPRT gene. nd: not detectable. (**B**) IL-6 and TNFα production was measured in supernatants by ELISA. (**C**) Time-course analysis of HMGB1 in the cytoplasm and nucleus of BMDCs not stimulated (NS) or stimulated by CpG 1826 (1 µg), *L. major* or vertebrate DNA (20 µg). ***(left)*** Analysis was performed by Western Blot at 30 and 60 min post-induction. ***(right)*** Quantification of HMGB1 in cytoplasmic extracts was normalized to that of actin and expressed as the n-fold difference with the unstimulated BMDCs. The mean and SEM of six independent experiments, each including both 30 min and 60 min extracts, is represented by the histogram. (**D**) C57BL/6 BMDCs were stimulated with *L. major* DNA alone (20 µg) or with glycyrrhizin (10 or 20 µg/ml) for 6 h. Percentage (%) of inhibition  =  [100-{cytokines production by *L. major* DNA with glycyrrhizin/cytokines production by *L. major* DNA alone}]×100. The data represent three independent experiments: one representative for C and the mean and SEM in A, B, D (*p<0,05, **p<0,01).

To determine whether HMGB1 could modify the immunostimulatory properties of DNA, BMDCs were stimulated either with *L. major* or vertebrate DNA alone, as well as with pre-formed HMGB1-DNA complexes. Stimulation with *L. major* DNA-HMGB1 complexes doubled cytokine mRNA expression and secretion compared with DNA alone (Figure 8A and 8B), whereas HMGB1-vertebrate DNA complex or HMGB1 alone did not. Similarly, no cellular stimulation was observed with sheep, pig or mouse DNA complexed with SLPI or LL37 peptides under these conditions (Figure S6). Therefore, enhanced DCs activation by SLPI, LL37, HMGB1 is only observed in the presence of parasitic DNA.

Activated immune cells, including dendritic cells, can secrete HMGB1 in response to various pro-inflammatory stimuli. Since HMGB1 increases the stimulatory activity of *L. major* but not that of vertebrate DNA, we wondered whether these two DNA could stimulate HMGB1 translocation from nucleus to cytoplasm in DCs. We showed here that the presence of extracellular DNA (CpG, *L. major* or vertebrate) was sufficient to promote a gradual release of HMGB1 from the nucleus to the cytoplasm: which is the first step of HMGB1 secretion. The accumulation of cytoplasmic HMGB1 reached a peak 30 min after stimulation with CpG and 60 min with the two eukaryotic D (Figure 8C). The absence of histone H3 in the cytoplasm indicated that cytoplasmic HMGB1 did not result from nuclear lysis. We next intended to evaluate whether extracellular forms of HMGB1 could mediate TLR9 activation in BMDCs exposed to parasite DNA. For this purpose cells were exposed to *L. major* DNA in the presence of glycyrrhizin, an inhibitor of extracellular HMGB1 [40]. Addition of this inhibitor reduced two-fold the DNA-triggered cytokine response (Figure 8D), confirming that the presence of extracellular HMGB1 contributed to BMDCs activation by parasitic DNA.

We next determined whether this effect could be due to the ability of HMGB1 to interact differentially with parasitic and vertebrate DNA. We tested this hypothesis with a gel retardation assay using sonicated DNA incubated with increasing amounts of HMGB1 protein passively transferred to a PVDF membrane.

**Figure 9:**
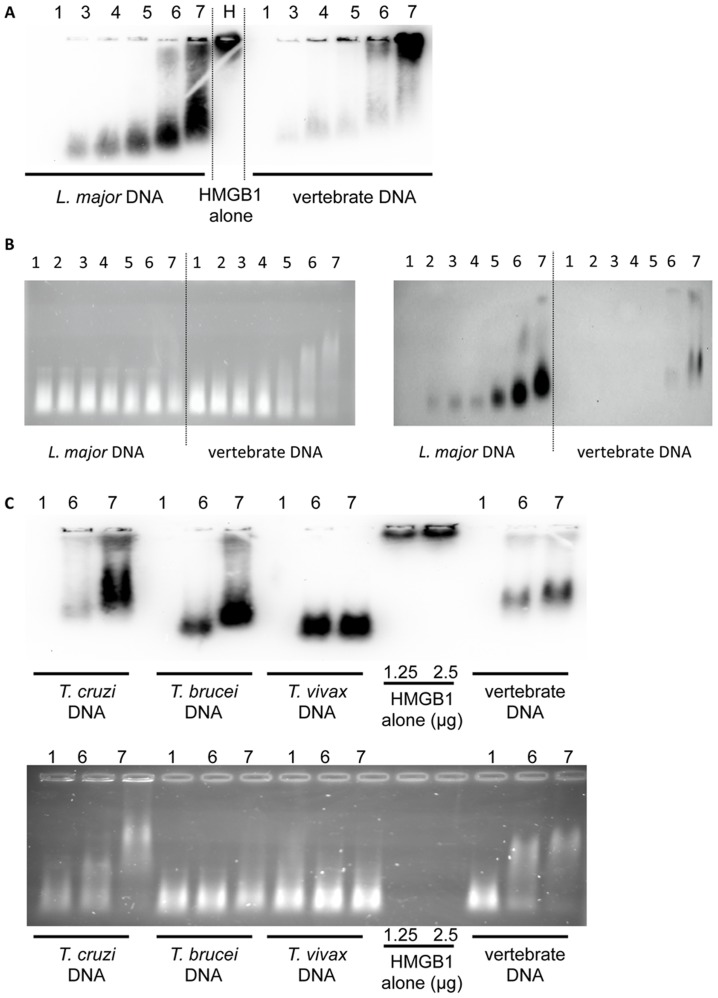
Analysis of DNA-HMGB1 complex by gel electrophoresis and Western blot. *L. major* or vertebrate sonicated DNA (250 ng) were incubated with increasing amounts of HMGB1. Lane 1 shows the migration of DNA alone and lane H the HMGB1 alone. In lane 2 to 7, DNA was complexed to HMGB1, respectively at a molar ratio of 2.5, 5, 7.5, 15, 25 and 50. (**A**) Following electrophoretic migration samples were immediately blotted on a PVDF membrane and revealed with an anti-HMGB1 antibody. (**B**) Following electrophoretic migration, the gel was incubated in a solution of EtBr during 45 min to stain the DNA ***(left)*** then blotted and revealed with anti-HMGB1 antibody ***(right)***. (**C**) Sonicated *Trypanosomatidae* DNA alone (250 ng) (lane 1) or complexed with HMGB1 at a molar ratio of 25 and 50 (lanes 6 and 7) were analysed by electrophoresis with the procedure described in **B**. Free HMGB1 (25 kDa) did not migrate in the 1% agarose gel.

As shown in Figure 9A, HMGB1 complexed with DNA had a different electrophoretic mobility than the HMGB1 alone (lane H), that did not migrate into the gel. Importantly, HMGB1 formed complexes with sonicated *L. major* DNA even for the lowest ratios of HMGB1/DNA, whereas it barely interacted with vertebrate DNA at the highest ratios. In HMGB1/*L. major* DNA lanes 3–7 (left part Figure 9A), little free HMGB1 is found, except at the highest molar ratios in lanes 6 (25∶1) and 7 (50∶1). In contrast, in HMGB1/vertebrate DNA lanes 3–7 (right part Figure 9A) a larger amount of free HMGB1 is observed in lanes 5 to 7. Quantification showed an increasing amount of bound HMGB1 on *L. major* DNA (from lane 3 to 7) while very low amount of HMGB1 was complexed with vertebrate DNA in the same conditions (Figure S7A). This result indicates that HMGB1 binding differs between -vertebrate and *L.major* DNA.

To highlight the size and electrophoretic pattern of complexed DNA (Figure 9B left), we subjected our gels to ethidium bromide (EtBr) staining before transfer. We found that increasing amounts of HMGB1 induce a dose-dependent retardation with both vertebrate and *L. major* DNA, albeit to a lesser extent. As observed earlier, higher amounts of HMGB1 are bound to *L. major* DNA (Figure S7B). In this condition, we no longer detect free HMGB1 with vertebrate DNA, suggesting that the small free HMGB1 proteins diffuse rapidly during the EtBr staining step (Figure 9B right).

Similar experiments were performed with *T. cruzi*, *T. brucei* and *T. vivax* DNA. Even though all sonicated *Trypanosomatidae* DNA have the same size, the DNA retardation profiles are different when DNA is bound with HMGB1 (Figure 9C). The amount of DNA bound HMGB1 depends on the nature of the parasite DNA and on HMGB1/DNA ratio (Figure 9C and Figure S7C). However, more HMGB1 is attached to *Trypanosomatidae* DNA than to vertebrate DNA, indicating that these *Trypanosomatidae* DNAs share the same property as *L. major* DNA.

Taken together, our results indicate that HMGB1 release by BMDCs is similar in response to parasite and vertebrate DNA. However, the propensity of HMGB1 to bind preferentially to parasitic DNA correlates with the increased activation of BMDCs in response to parasitic DNA.

## Discussion

While TLR9 shares a common function of nucleic acid recognition along with other TLRs (TLR3, TLR7 and TLR8), it is also involved in parasite recognition [1], [2], [4], [8], [41], [42]. Previously, it has been reported that DNA from certain protozoan parasites (*B. bovis*, *T. cruzi*, *T. brucei*) stimulated B cell proliferation and macrophage activation [1], [43], [44]. Here we provided experimental evidences that account for the specific activation of TLR9 pathway by *L. major* and other *Trypanosomatidae* DNA, which is not the case of vertebrate DNA.

Previously, it has been proposed that the discrimination between microbial and self-DNA is based on the endosomal localization of TLR9 and the failure of self-DNA to access endosomes [16], [18]. Here we have demonstrated that the minimal immunostimulary activity by vertebrate DNA compared to *L. major* DNA is not due to a limited accessibility for TLR9 since we observed the same uptake rates for both DNAs in BMDCs. By enhancing DNA translocation in endosomes with DOTAP, there was a significant increase in DCs activation by *L. major* DNA that remained TLR9-dependent while there was a low cellular activation only by a larger amount of complexed vertebrate DNA. In quantitative terms, four times more vertebrate DNA complexed to DOTAP displays 10% of the stimulatory activity of *L. major* DNA. Therefore, the same amount of DNA taken up by BMDCs may be sufficient to cause cellular activation by parasite DNA, but not by vertebrate DNA. We also eliminated the possibility that the lack of activation by vertebrate DNA was due to its large genomic size, as the differences in the BMDCs activation between *L. major* and vertebrate DNA persisted when the DNA were reduced to the same size by sonication.

DNA-binding protein and cofactors such as UNC93B1 are also implicated in the endosomal TLR9 recognition. UNC93B1 mutant mice are highly susceptible to *L. major* and to *T. cruzi*, showing the involvement of TLR9 but also TLR7 and TLR3 in resistance to *L. major* infection [41], [42]. Our work sheds light on the involvement of auxiliary protein associated with *L. major* DNA in TLR9 activation. We observed an increase in the expression of proinflammatory cytokines in DCs activated by *L. major* DNA complexed with different peptides as LL37, SLPI or HMGB1. We assumed a potential role of HMGB1 in the specific stimulation of *L. major* DNA by BMDCs, since HMGB1 increases the recognition of CpG-ODN by TLR9 and extracellular HMGB1 accelerates its delivery to the receptor [22], [29]. Indeed, we have observed an increase in the expression of cytokines in stimulated BMDCs by HMGB1 complexed to *L. major* DNA but not when HMGB1 is alone or complexed to vertebrate DNA. In resting immune cells, HMGB1 is highly abundant in the nucleus but shuttles between it and the cytoplasm [45]. Following the activation by *L. major* and vertebrate DNA, the shuttle system is disturbed and HMGB1 gradually accumulates in the cytoplasm before being eventually secreted as described by Ivanov, after CpG stimulation of BMDCs. The extracellular contribution of HMGB1 was proved herein by the fact that glycyrrhizin [40], a known inhibitor of extracellular HMGB1, decreased cytokine production in response to DNA. HMGB1 may interact with DNA out of the cell, to eventually act as a co-stimulating factor. However we may also consider that this interaction take place within the cell, as suggested by the rapid cellular uptake of DNA and the subsequent translocation of HMGB1 after DNA activation.

To define more precisely HMGB1 activity at the molecular level, we compared the interaction of HMGB1 with both DNAs. Although HMGB1 could interact with both DNAs *in vitro*, there was surprinsingly more HMGB1 on *L. major* than on vertebrate DNA. Similar observations were made for other *Trypanosomatidae* DNA (*T. cruzi*, *T. vivax*, *T. brucei*). This strongly suggested that intrinsic differences between vertebrate and parasitic DNA might favour HMGB1 binding to parasitic DNA, therefore enhancing its contribution through TLR9-dependent pathways. Obviously, it is tempting to associate HMGB1 binding for parasitic DNAs to their composition and/or structure. First, *L. major* DNA as other *Trypanosomatidae* DNA is composed of a nuclear and a kinetoplastic DNA, consisting of a network of particular DNA structures (maxi and mini-circles) [46]. HMGB1 is considered to be a non specific single- or double-stranded DNA binding protein with special affinity for distorted DNA structures such as supercoiled DNA or DNA minicircles [27]. Secondly, the *L. major* genome is more GC rich than the mouse or the human genome (63% against 42%). Interestingly, one report described that HMGB1 has some preference for binding CpG-rich oligonucleotides over GpC/GpG ODNs on single-stranded DNA [29]. HMGB1 has also been reported to preferentially bind to stable and high-ordered structures as G-tetrads [47], resulting from polyG sequences, which reduces nuclease sensitivity [37].

*L. major* DNA is more resistant to both nucleases, DNases I and II, than vertebrate DNA. DNase I is a nuclease responsible for degrading extracellular DNA. Dnase II is an ubiquitous lysosomal endonuclease that requires an acidic environnement to cleave DNA [36]. Our experiments suggest that the parasitic DNA could persist longer in the cell and therefore act as a better activator for TLR9. Besides, this correlates with the higher proportion of polyG sequences in *L. major* DNA than in mouse DNA. Despite its greater sensitivity to DNases, vertebrate DNA was proved to inhibit TLR9 activation by *L. major*. This implies that the differences found between vertebrate and *Trypanosomatidae* DNA are due to their intrinsic properties and their nucleotidic motifs.

Initially, TLR9 was identified as the receptor for oligonucleotides containing unmethylated CpG motifs [9]. It has been proposed that *Trypanosomatidae* DNA (*T. cruzi, T. brucei*) are hypomethylated and stimulate the expression of inflammatory cytokines [4], [43]. Overmethylation of *T. cruzi* and *T. brucei* reduced but did not eliminate the stimulatory activity of these *Trypanosomatidae* DNA. Moreover, it has been found that even predominantly or completely unmethylated DNA was still not stimulatory [9], [14]. Therefore, the idea that stimulatory properties of DNA correlate solely with the presence of unmethylated CpG motifs may be an oversimplification [14]. This implies that vertebrate DNA could contain unknown structural motifs that inhibit the immunostimulatory function of its unmethylated CpG motifs.

More recently, it has been shown that the DNA sugar backbone 2′deoxyribose represents a prime determinant for the interaction between single-stranded DNA and TLR9. In its natural phosphodiester state, the base-free 2′deoxyribose backbone acts as a basal TLR9 agonist and the addition of DNA bases, even lacking CpG motifs, enhances its agonist activity [11]. This suggests that any mammalian or pathogen DNA could activate TLR9. However, along with other works [10], [13], we have shown that naked vertebrate DNA fails to activate innate immune cells.

Besides, it has been reported that both stimulatory and inhibitory DNA oligonucleotides can interact with TLR9, which also suggested a non specific process of recognition, but only stimulatory oligonucleotide could induce conformational changes leading to MyD88 recruitment and TLR9 signaling [48]. Competition for TLR9 activation has been already observed between CpG and inhibitory oligonucleotides [10], [49], [50]. In the presence of vertebrate DNA, we observed a similar inhibition of the activation induced by *L. major* DNA. Thus, vertebrate DNA could also be an effective TLR9 ligand. Altogether the data mentioned above on the differences between *L. major* and vertebrate DNA, with respect to HMGB1 interaction, resistance to DNase and DNA competition for TLR9 signaling, led us to compare their genomic sequences. Canonical and non canonical stimulatory and inhibitory sequences were investigated in both types of genome. The ratio of observed to expected (rO/E) stimulatory sequences is on average five times more in *L. major* DNA than in mouse and human DNA, whereas the ratio for inhibitory sequences is on average two times lower. This suggests that vertebrate genomes have counter-selected stimulatory motifs and selected for inhibitory motifs, presumably to avoid auto-immunity and/or better discriminate non-self DNA [18]. These differences in their sequence are moreover consistent with the observed competition between *L. major* and vertebrate DNA. Further analysis of different *Trypanosomatidae* genomes confirmed the presence of more stimulatory and less inhibitory sequences, compared to vertebrate genomes. Inhibition by vertebrate DNA can be generalized to other *Trypanosomatidae* DNA since it is based on a competition between DNA motifs contributing to TLR9 activation or inhibition.

Until now very few data were available regarding nucleotide sequence and cellular events involved in the differential recognition of parasite and vertebrate DNA by TLR9. Interestingly, the stimulatory activity of *T. cruzi* DNA is correlated with the finding that mouse- and human- like CpG motifs for TLR9 are clustered on retrotransposon VIPER (vestigial interposed retroelement) elements and mucin-like glycoprotein genes in the *T. cruzi* genome [4]. However, *in L. major* DNA, the stimulatory motifs are distributed throughout the whole genome and not concentrated in particular genomic regions as for *T. cruzi*.

As it has been demonstrated by Krieg et al, 1998, with adenovirus DNA that some serotypes are immunostimulatory and other not, due to differences in stimulatory or neutralizing CpG, we are convinced that the disbalance between stimulatory an inhibitory sequences could explain why *L. major* is a potent activator DNA, in comparison of vertebrate DNA. Moreover we agree with the data from Stacey et al, 2003, wich suggested that a low frequency of active CpG may never reach sufficient concentration within the cells to cause cellular activation.

It is interesting to note that human TLR9 also recognizes *L. major* DNA, leading to TLR9 signaling, but not the self-DNA. This ability of TLR9 to discriminate pathogen DNA from the self DNA is mentioned as a lock for additional security, enabling the cell to maintain its integrity [18].

This work brings further insights into how TLR9 discriminates between *Trypanosomatidae* and vertebrate DNA. We show that DNA sequences in *Trypanosomatidae* trigger activation of TLR9. Additionally, we show for the first time the involvement of HMGB1 in the reponse to *L. major*. This result suggests that the interaction of parasite DNA with DNA-binding protein is involved in TLR9 signaling and, thus, in the innate immune response to this parasite.

## Supporting Information

Figure S1 **Stimulation of C57BL/6 and TLR9-/- BMDCs by** ***Trypanosomatidae*** **DNA, LPS or CpG.** BMDCs from C57BL/6 and TLR9-/- mice were stimulated 6 h with *Trypanosomatidae* DNA (20 µg), LPS (100 ng/ml) or CpG (0.25 µg/ml) and cytokines production was analysed by PCR. The data represent the mean and SEM of three independent experiments. nd: not detectable.
(TIF)

Figure S2 **Activation of BMDCs by complexed vertebrate DNA with DOTAP.** (**A**) BMDCs from C57BL/6 mice were stimulated *in vitro* 6 h with vertebrate DNA (from mouse or pig: 20 µg/ml) alone or complexed with DOTAP (10 µg/ml). IL-6 and TNFα production was analysed by ELISA. The data represent the mean and SEM of three independent experiments. (**B**) Sonicated mouse and L.major DNA size were analysed by electrophoresis and EtBr staining.
(TIF)

Figure S3 **Characterization of the Gen2.2 plasmacytoid dendritic cell line.** (**A**) Cell surface expression of CD11c, CD45RA, HLA-DR and CD123 on Gen2.2 was analysed by flow cytometry under resting condition. Filled histograms represent staining with isotype control. (**B**) Cytokines mRNA were detected by real time PCR after stimulation of Gen2.2 cells stimulated for 6 h with CpG-A (5 µg) or with the imidazoquinoline Cl-097 (0.25 µg). Results are from three independent experiments.
(TIF)

Figure S4 **Sonicated vertebrate DNA and inhibitory oligonucleotide, but not LPS, inhibit** ***L.major*** **DNA stimulation.** BMDCs from C57BL/6 mice were stimulated 6 h with full-length (FL) L.major DNA (10 µg) alone or with sonicated (SC) vertebrate DNA (10 µg) or inhibitory oligonucleotide (ODN) 2088 (1 µg) or with LPS (100 ng/ml). Cytokines production was analysed by ELISA. Results are from one of two independent experiments.
(TIF)

Figure S5 **BMDCs stimulation by** ***L.major*** **DNA complexed with different cofactors.** BMDCs were stimulated with *L.major* DNA complexed with SLPI (20 µg/ml), LL37 (2 µg/ml), or HMGB1 (1 µg/ml) or alone (as control) for 6 h. Expression of indicated cytokine was determined by real time PCR. The data are expressed as the n-fold difference with the expression in stimulated BMDCs by *L. major* DNA alone. The mRNA expression levels were normalized to the expression of the HPRT gene. Results are from one of three independent experiments.
(TIF)

Figure S6 **BMDCs stimulation by complexed vertebrate DNA with different cofactors.** BMDCs were stimulated with vertebrate DNA (mouse, pig or sheep: 20 µg/ml) complexed with SLPI (20 µg/ml), LL37 (4 µg/ml), or HMGB1 (1 µg/ml) or alone for 6 h. IL-6 and TNFα production was mesured by ELISA in the supernatants of stimulated BMDCs for 6 h. The data represent the mean and SEM of three experiments.
(TIF)

Figure S7 **Quantification of** ***Trypanosomatidae*** **and vertebrate DNA bound HMGB1.** Quantifications in A, B, C were performed respectively on the western blots submitted in Figures 9A, 9B right, 9C down using ImageJ software. In lane 2 to 7 sonicated DNA (250 ng) was complexed to HMGB1, respectively at a molar ratio (rHMGB1/DNA) of 2.5, 5, 7.5, 15, 25 and 50. *T.cruzi, T.brucei, T.vivax* DNA were complexed to HMGB1 at a molar ratio (rHMGB1/DNA) of 25 and 50.
(TIF)

Table S1 **Characteristics of** ***Trypanosomatidae*** **and vertebrate genomes.** The left part shows the genomic size and the frequency for each nucleotide. The right part shows the observed numbers of motifs ((GGGG)_2_, GACGTT, GTCGTT, TTAGGG), counted by the in-house computer program (wcount). *nc: not counted*.
(DOC)
