# Supplementary figures and images for: TLR9 Activation Is Triggered by the Excess of Stimulatory versus Inhibitory Motifs Present in Trypanosomatidae DNA
Source: PLoS Negl Trop Dis. 2014 Nov 13;8(11):e3308. doi: 10.1371/journal.pntd.0003308 (PMC4230925; doi:10.1371/journal.pntd.0003308)

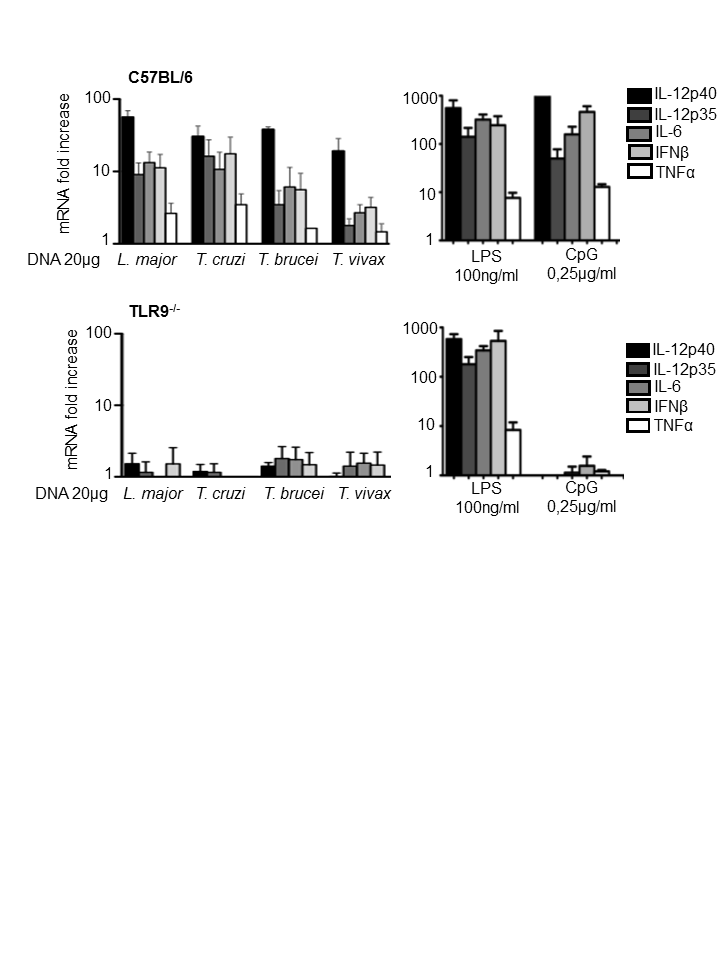

Supplement: Figure S1 — Stimulation of C57BL/6 and TLR9-/- BMDCs by Trypanosomatidae DNA, LPS or CpG. BMDCs from C57BL/6 and TLR9-/- mice were stimulated 6 h with Trypanosomatidae DNA (20 µg), LPS (100 ng/ml) or CpG (0.25 µg/ml) and cytokines production was analysed by PCR. The data represent the mean and SEM of three independent experiments. nd: not detectable. (TIF) [file pntd.0003308.s001.tif]

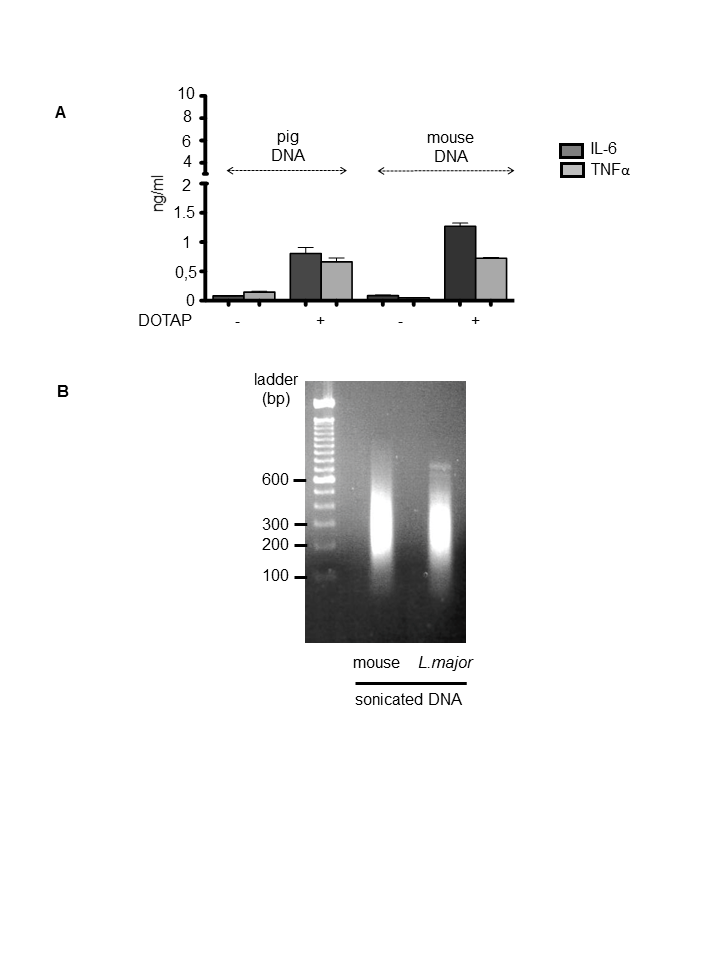

Supplement: Figure S2 — Activation of BMDCs by complexed vertebrate DNA with DOTAP. (A) BMDCs from C57BL/6 mice were stimulated in vitro 6 h with vertebrate DNA (from mouse or pig: 20 µg/ml) alone or complexed with DOTAP (10 µg/ml). IL-6 and TNFα production was analysed by ELISA. The data represent the mean and SEM of three independent experiments. (B) Sonicated mouse and L.major DNA size were analysed by electrophoresis and EtBr staining. (TIF) [file pntd.0003308.s002.tif]

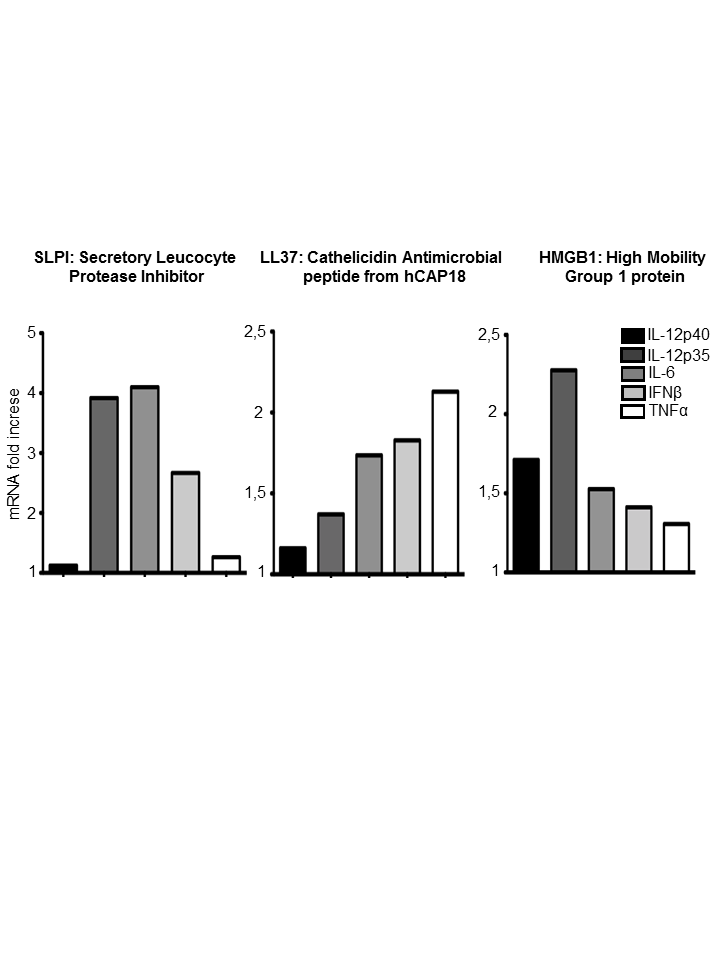

Supplement: Figure S5 — BMDCs stimulation by L.major DNA complexed with different cofactors. BMDCs were stimulated with L.major DNA complexed with SLPI (20 µg/ml), LL37 (2 µg/ml), or HMGB1 (1 µg/ml) or alone (as control) for 6 h. Expression of indicated cytokine was determined by real time PCR. The data are expressed as the n-fold difference with the expression in stimulated BMDCs by L. major DNA alone. The mRNA expression levels were normalized to the expression of the HPRT gene. Results are from one of three independent experiments. (TIF) [file pntd.0003308.s005.tif]
