# Supplementary material for: TLR9 Activation Is Triggered by the Excess of Stimulatory versus Inhibitory Motifs Present in Trypanosomatidae DNA
Source: PLoS Negl Trop Dis. 2014 Nov 13;8(11):e3308. doi: 10.1371/journal.pntd.0003308 (PMC4230925; doi:10.1371/journal.pntd.0003308)
Supplement: Table S1 — Characteristics of Trypanosomatidae and vertebrate genomes. The left part shows the genomic size and the frequency for each nucleotide. The right part shows the observed numbers of motifs ((GGGG)2, GACGTT, GTCGTT, TTAGGG), counted by the in-house computer program (wcount). nc: not counted. (DOC) [file pntd.0003308.s008.doc]

|  | **Genome** | **Nucleotide frequency** | | **Copy number/10^6^bp** | | | |
| --- | --- | --- | --- | --- | --- | --- | --- |
|  | **size** (Mb) | **A/T** | **C/G** | **(GGGG)_2_** | **GACGTT** | **GTCGTT** | **TTAGGG** |
| ***L. major*** | 32.8 | 0.20 | 0.30 | 414 | 183 | 271 | 121 |
| **Mouse** | 2760 | 0.29 | 0.21 | 113 | 33 | nc | 205 |
| **Human** | 3220 | 0.29 | 0.21 | 11 | nc | 21 | 168 |
| ***T. cruzi*** | 32.5 | 0.24 | 0.26 |  |  |  |  |
| ***T. brucei*** | 26.5 | 0.27 | 0.23 |  |  |  |  |
| ***T. vivax*** | 22.7 | 0.23 | 0.27 |  |  |  |  |
